# The role of digital transformation in addressing health inequalities in coastal communities: barriers and enablers

**DOI:** 10.3389/frhs.2023.1225757

**Published:** 2023-08-30

**Authors:** Sheena Asthana, Samantha Prime

**Affiliations:** Centre for Health Technology, University of Plymouth, Plymouth, United Kingdom

**Keywords:** digital health technologies (DHTs), variations in digital maturity, NHS (National health service), coastal health inequalities, the quadruple aim of health systems, shifting the balance from cure to prevention

## Abstract

Healthcare systems worldwide are striving for the “quadruple aim” of better population health and well-being, improved experience of care, healthcare team well-being (including that of carers) and lower system costs. By shifting the balance of care from reactive to preventive by facilitating the integration of data between patients and clinicians to support prevention, early diagnosis and care at home, many technological solutions exist to support this ambition. Yet few have been mainstreamed in the NHS. This is particularly the case in English coastal areas which, despite having a substantially higher burden of physical and mental health conditions and poorer health outcomes, also experience inequalities with respect to digital maturity. In this paper, we suggest ways in which digital health technologies (DHTs) can support a greater shift towards prevention; discuss barriers to digital transformation in coastal communities; and highlight ways in which central, regional and local bodes can enable transformation. Given a real risk that variations in digital maturity may be exacerbating coastal health inequalities, we call on health and care policy leaders and service managers to understands the potential benefits of a digital future and the risks of failing to address the digital divide.

## Introduction

There is growing acknowledgement that, with respect to both crude (unadjusted) and standardised measures, England's coastal communities have significantly higher needs for NHS, social care and public health services than their inland counterparts. The Chief Medical Officer's (CMO's) 2021 annual report highlighted the substantially higher burden of physical and mental health conditions in coastal communities, often with lower life expectancy. This is partly explained by the fact that that coastal populations tend to be both older and more deprived than non-coastal populations. However even after adjusting for these factors (and others including ethnicity), there still appears to be a “coastal excess” in the prevalence of disease and risk factors (1).

There is also evidence of a significant health service deficit in terms of recorded service standards, cancer indicators and emergency admissions in coastal communities. Health Education England's analysis for the 2021 CMO report found that, despite coastal communities having older and more deprived populations, they have 14.6% fewer postgraduate medical trainees, 15% fewer consultants and 7.4% fewer nurses per patient (2). Reasons for the mismatch between workforce and disease prevalence in coastal areas are not understood. However, it is worth noting longstanding concerns that, due to the inherent circulatory of activity-based resource allocation models, systematic biases introduced in the 2002 AREA formula (3) may still be disadvantaging areas serving older demographic populations. There are also concerns that NHS funding formulae do not adequately capture the difficulties providers face in achieving economies of scale, as their coastal location gives them a 180-degree (at best) catchment area (4) and the populations they serve tend to be geographically dispersed.

With no fundamental changes to the system of NHS resource allocation in sight, challenged health and care systems in coastal areas need to start working differently. In this paper, we propose that, by shifting the balance of care (5) from reactive to preventive, supporting early diagnosis and care at home and using advanced data analytics and Artificial Intelligence (AI) to improve clinical workflows, digital transformation has the potential to promote the “quadruple aim” (6) of better population health and reducing inequalities, improved experience of care, healthcare team well-being *and* lower system costs. However, there are a range of barriers to realising this potential, including variations in digital maturity which tend to disadvantage coastal areas. We discuss these barriers and identify strategies that policy leaders and health service managers can deploy to enable digital transformation.

## The coastal excess

The “coastal excess” in disease burden in England largely reflects the fact that coastal populations tend to be both older and more deprived than non-coastal populations. 16.6% of coastal residents live in one of the 10% most deprived Lower Super Output Areas (LSOAs) in the country, compared with 8.4% of non-coastal residents. At the other end of the scale, 5.1% of coastal residents live in one of the least deprived LSOAs, compared with 10.6% of people living in non-coastal areas. Moreover, although the proportion of people aged 65 and above varies with deprivation, it is always far higher in coastal areas. Overall, 21.0% of people living in coastal LSOAs are aged 65+, compared with 17.8% in non-coastal LSOAs.

With both age and deprivation associated with an increased risk of disease, it is hardly surprising that analysis carried out for the CMO 2021 report found higher prevalence rates of a range of diseases in coastal communities (1), the coastal excess of cardiovascular diseases and chronic obstructed pulmonary disease (COPD) being particularly stark. However, age and deprivation did not fully account for the difference. Indeed, once age, socio-economic status and ethnicity were accounted for, COPD and mental health prevalence rates were found to be nearly 11% higher in coastal than non-coastal communities. There was also evidence of very poor outcomes for children and young people, with high rates of hospitalisation for self-harm, alcohol and substance use. Reflecting a shift in the distribution of children living in poverty since the 1990s, this may be an early indicator of a future public health crisis in these communities (7).

The reasons for this coastal excess are not fully understood. However, research points to the emergence of new and worrying patterns of deprivation and associated complex inter-linked challenges amongst “lagging” coastal communities, including high levels of unemployment, low incomes, seasonal jobs, low skills and poor educational outcomes, detrimental patterns of selective in- and out-migration, unaffordable housing, hidden homelessness and high rates of anti-depressant and opioid prescribing (8–15). Economic decline and socio-economic deprivation in coastal areas exacerbate the risk of developing non-communicable diseases (NCDs), particularly at a younger age (i.e., before 65 years old). Low pay and low job security reduce access to material resources such as housing and healthy food and increase exposure to occupational hazards (16). Low job status with less autonomy and income insecurity are also key risk factors for chronic psychological distress (17, 18), a risk factor for chronic inflammation and in turn the development of NCDs (19, 20). As noted above, a significant excess has been found in coastal LSOAs with respect to poor mental health.

Insofar as they are known risk factors for poor parenting and associated impacts on children's development, financial insecurity and poor parental mental health may be one of many factors behind the increasing gap in health inequalities among children and young people between the core and periphery. Education can provide a protective effect in the longer run. Predicting employment, income and access to material resources as well as psychosocial well-being and health behaviours, education is arguably the single most important modifiable social determinant of health (21). Various mechanisms have been proposed (22, 23). First, education leads to better-paid and more stable jobs with greater autonomy and less exposure to psychosocial stress. Second, there is growing evidence that education plays a direct role in developing psychological resilience (24). Resilience has been identified as a predictor of health in children (25, 26) and adults (27, 28), possibly through protection from stress-induced immune changes (29–31). A third causal pathway linking education, resilience and health is self-efficacy, a concept that refers to an individual's belief in their ability to exert control over their health risks behaviours. Against this background, the fact that educational performance is significantly worse in the coast then in inland areas should be a cause for public health concern.

## The role of digital transformation in shifting the balance of care

A vast array of digital health technologies (DHTs) exist that can prevent ill-health, promote well-being and support the delivery of health and social care. This includes smartphone apps, wearable devices (transformed to THINKables when data are analysed at the point of sensing to inform real-time responsiveness), smart home devices and environmental sensors, virtual reality, surgical and care robotics and platforms that provide remote healthcare (telehealth). As the uptake of such technology increases, the potential of artificial intelligence (and associated machine learning and national language to monitor individual patient risk, design intelligent triggers and support health care decision making will also escalate.

It is not within the scope of this paper to provide a comprehensive review of the potential of DHTs to support the “quadruple aim”. Instead, we highlight areas where there are likely to be technological solutions to key coastal challenges.

### Digital mental health interventions

Among children and young people (CYP) who generally enjoy good physical health, mental health problems are a particular concern, in part because of their relatively high prevalence, in part because of their impact on future developmental trajectories, including risk of premature NCDs. As noted above, CYP in coastal areas are showing higher prevalence rates of mental distress. Could DHTs offer a solution to managing this higher burden?

As we discuss below, one of the challenges to assessing the role of DHTs in health prevention and promotion is that relatively few are “evidence based” (i.e., subject to sufficiently rigorous evaluation with respect to their effectiveness and particularly cost-effectiveness). One of the few systematic reviews in this area found some support for the clinical benefit of DHTs, particularly computerised cognitive behavioural therapy for depression and anxiety in adolescents and young adults but uncertain evidence about the benefits for managing attention deficit/hyperactivity disorder, autism, anxiety, depression, psychosis, eating disorders and post-traumatic stress disorder (32). Indirectly, DHTs may promote young people's health chances by e.g., mediating parenting behaviours (33). Plausibly, more effective innovations for coastal youth would address the complex interlinkages between poor employment prospects, low aspirations, poor mental health and the potential of e.g., outreach from metropolitan-based corporations to both mentor and offer on-line job opportunity experience. We are not aware of either initiatives focused on coastal outreach or evaluation evidence to support investment in this approach.

Uncertain evidence about the benefits of DHTs needs to be balanced against known concerns about the current state of Child and Adolescent Mental Health Services (CAMHS) in the UK, which has been overly medicalised (most investment going into health as opposed to preventive education and community services), operates a high threshold for referral—prior to the backlogs exacerbated by Covid19, only 25% of children or young people referred to specialist CAMHS were seen (34) and which are now subject to waiting times that commonly exceed one year. Higher levels of digital engagement by children and young people also suggest that DHTs may offer a unique opportunity to address unmet needs. Against this background, this may a fruitful area for practitioners and policy makers in coastal areas to explore.

Robust evidence of the effectiveness of DHTs in improving the mental health of adults is similarly lacking. Again, in the context of a shortage of mental health provision, there is growing interest in the potential of AI-based chatbots, avatar therapy and companion bots (e.g., using conversational applications) in managing psychiatric symptoms and augmenting therapeutic treatments (35). A failure to meet evidential requirements together with clinician concerns about safety, accountability and legal responsibilities (36) have limited the implementation of such technologies into current care pathways. A balance may need to be struck in highly challenged health care systems between risk aversion and an openness to exploring the potential of innovation in ways that are methodologically robust but, in the context of rapidly evolving DHTs, may not be conducive to classic randomised control trial (RCT) methodologies, a point to which we return below. Again, considering the role of DHTs within the vision of addressing the wider determinants of health, more research needs to be conducted on the potential of digital technologies to promote self-efficacy, capabilities and well-being in a more holistic understanding of people's lives as opposed to narrow patient activation measures.

## Digital support for primary and secondary prevention

The evidence base for DHTs is more established with respect to secondary prevention (where people with known risk factors for disease or existing conditions have been selected for e.g., remote monitoring) than for primary prevention. For example, the use of wearables such as Apple watches to monitor a range of measures from physical activity, heart rate, blood pressure, sleep patterns to fertility patterns is defined as “consumer” (i.e., unregulated) health care and often strictly separated from DHTs that are deemed medically safe. Insofar as such devices offer the opportunity to exploit continuous as opposed to intermittent data in diagnosis and management, there may be a case for being more flexible about incorporating data from consumer devices into health care records.

In contexts (generally outside the UK) that allow the real-time transfer of physiological data from wearables to electronic patient records, there is evidence of benefit to e.g., elderly care generally (37), as well as supporting the early diagnosis of deterioration in e.g., cardiovascular, neurological and pulmonary diseases and the identification (e.g., through AI of triggers for rapid intervention (38, 39).

Smart devices and environmental sensors can also facilitate the remote monitoring of the home environment (40) (such as temperature, humidity, and smoke) as well as important physiological signs and activities (41) of occupants, supporting functions from the simple (e.g., alarms for critical event alerts) to the more complex identification of anomalies in usual behaviours (42), including diet, physical activity and social interaction through, e.g., acoustic sensing, Radio Frequency sensing and passive environmental sensing. Novel data science and machine learning (ML) techniques offer enormous potential for analysing such data and improving safe care in the home including, if necessary, from a distant facility.

Virtual reality (VR) is increasingly used in healthcare as an educational tool. Hardware limitations of headsets have raised questions about the practicality of prolonged use by vulnerable or frail patients, though recent advances in mobile-based light-weight headsets have been shown to be feasible to deploy in challenging healthcare settings. There are now plausible studies demonstrating the potential value of VR in supporting a range of physical, mental, or psychosocial health outcomes (43), including pain management (44), cognition and depression (45).

Care robotics are an emerging technology in the UK and can support independence for both adults and children as well as addressing staff shortages. When robots are equipped with AI and depth cameras, they can monitor patients as they go through prescribed exercises, tracking progress more precisely than the human eye. Social robots can interact adaptively (46) with patients to provide coaching, encouragement and monitoring, including in long-term care environments for older adults (47) and those with dementia (48). They may encourage patients to comply with treatment regimens or provide cognitive engagement, though again, more research has been done on the internal validity of social robots than the factors that affect their implementation in real-world practice.

There are also numerous applications of AI/ML, including risk stratification in population health management, AI-based platforms leveraging real world data to provide insights as to which patients can benefit from home-based technologies to manage the risk upfront to lower downstream costs. Voice assistants (49) can be used to set simple reminders to e.g., take medicines. As the uptake of wearables, personal devices and sensors increases, the potential of AI based technologies to monitor individual patient risk and design intelligent triggers will also escalate.

### The smart hospital

As with supporting prevention, early diagnosis and management at home, there are numerous types of technologies that can transform the quality and efficiency of care within the hospital environment including: location recognition and tracking technology, coordination and communication services, Internet of Things-based technology, mobile devices and wearables, telehealth, AI, robotics and extended reality and 3D printing (50). Further, the rise of intelligent buildings in health and social care such as smart hospitals, will accelerate digital technologies deployed specifically to support the entire lifecycle of estates as part of Building Management Systems and Building Information Modelling (BIM) processes.

Though lacking in formal definition, notionally, smart hospitals are born when a matrix of digital solutions converge in the real world through their considered integration into the fabric, footprint and flow of healthcare facilities—this includes in-flow, within flow and outflow. Ultimately such a convergence of technology and associated data will bring with it an embodied intelligence capable of improving patient outcomes and experiences, staff satisfaction and retention, operational efficiencies and environmental sustainability. While intelligent buildings are commonplace in the commercial sector, there remains a paucity of guidelines specific to health and care facilities for which managers and decision makers can follow. In large part this is due to the considerable variation in contextual nuance such as needs, resources, policies and practices. Importantly, smart hospitals are not a status to be attained *per se*, but a concept heavily influenced by contextual facture such as the baseline digital maturity of a facility and region as well as the innovation and transformation appetite and opportunity. Major healthcare estates projects, particularly greenfield constructions or redevelopments, present significant opportunities to accelerate digital adoption. Not least because they allow for technology to be incorporated into the fabric of the building in ways previously unattainable thanks to modern methods of construction, but also because they allow for core digital infrastructure to be architected from inception to allow facilities to avail themselves of contemporary technologies—including those yet to be conceived.

Practical examples of technology within hospitals include robots which are increasingly used to assist in surgical procedures, manual handling, and to supplement and enhance cleaning [e.g., using Ultraviolet light (51)], perform routine (e.g., transporting) tasks and support patients with anxiety and rehabilitation. However, within the published literature, much of the focus has been on the use of advanced data analytics and AI in conjunction with Electronic Patient Records (EPRs) to improve in-patient outcomes and, by supporting an optimal allocation of resources (including staff resources in response to predicted demand), cost-effectiveness.

The American EPR, EPIC, is the most studied system internationally, in part because it holds the current leading market share (29%) in the US, where studies have demonstrated improvements in both clinical outcomes and efficiency (52). The example of just one of its 30 “Cognitive Computing” (ML) models, Clinical Deterioration, demonstrates the potential of improving prediction in ways that support patient outcomes as well as overall costs.

Approximately 5%–10% of hospitalised patients encounter a severe adverse event during their hospital admission, including cardiac arrests and admission to an intensive care unit. Delays in the recognition of deterioration lead to delayed diagnostic and therapeutic interventions resulting in increased morbidity and mortality and associated costs. Since 2012, acute hospitals in the NHS have used the National Early Warning Score (NEWS) to detect acute illness severity and clinical deterioration (53). This is based on a simple aggregate scoring system in which a score is allocated to physiological measurements (respiration rate, oxygen saturation, systolic blood pressure, pulse rate, level of consciousness or new confusion, temperature) already recorded in routine practice when patients are present or being monitored in hospital. The EPIC Clinical Deterioration AI model, currently in use in 12 countries (but not in the UK) comprises a far wider range of variables and uses trained models to predict risk of critical illness and clinical deterioration in hospitalised patients. US studies using the EPIC clinical deterioration model have shown a dramatic improvement when the model was used in comparison to the US equivalent of NEWS. This is but one example of the possibility of replacing a simplistic and largely reactionary system with a proactive, predictive approach. However, the reasons why EPIC AI models are not being deployed in any of the UK hospitals that have procured the EPR shed light on the complex barriers to unlocking the potential of DHTs in the NHS and most particularly in coastal areas.

## Barriers to digital transformation

### Variations in infrastructural readiness

To make the most of digital innovations, we need platforms for the real-time transfer of data from wearables, sensors etc. to EPRs. Despite a strong policy push and the catalyst of the COVID-19 pandemic, the NHS has had variable success with embedding a digital infrastructure (54) and has consequently struggled to adopt eHealth innovations. Recent research for the Health Services Journal reveals that, in 2022, 27 acute trusts in England did not even have comprehensive EPRs (55). While some of these use smaller-scale electronic systems in individual departments, several continue to rely on largely paper-based patient records. It is important to note that hospital trusts in coastal and rural areas on England's peripheral are over-represented among this group, reflecting previous investment, e.g., in Local Health and Care Record Exemplars (LHCREs)and Digital Innovation Hubs, that benefitted only areas that were already judged to be “digitally mature”. Areas such as Greater Manchester and London are now galloping ahead with respect to their digital health ecosystems and, as such, attracting further investment from both Government and industry. As a result, digital maturity would appear to be geographically concentrating rather than trickling down (54).

While other parts of the health and care system have EPRs (in England, for example, general practice is universally covered), the absence of a system within large acute hospitals tends to be associated with a lack of data integration in the wider local ecosystem. Innovative general practitioners (GPs) may be monitoring their patients with e.g., wearables. However, in most cases, they will have had to purchase Application Programming Interfaces (APIs) to link programme software to patient records and data will be manually monitored. This is manageable for small groups of patients. However, to scale up, data need to be integrated into an orchestration layer (coordinating communication and interaction between APIs, as well as managing data flow and security) and, for large datasets, processed using advanced and automated data analytics. The goal would be to have a centralised “command and control centre” that can monitor system demand and have processes to proactively respond to it, e.g., by alerting responders (emergency, community, social and voluntary services as well as GPs and carers) to suitably calibrated (and transparent) triggers and to regulated responders. At present, large swathes of England have no (or at best rudimentary) orchestration platforms and there is systematic (coastal vs. non-coastal) variation in infrastructural readiness.

Indeed, in many areas work is still in progress linking primary, secondary and other data into “shared care records”, the integration of which tends to be more advanced in LHCRE areas than in those that lacked LHCRE status and have pursued a “minimum viable solution”. There is growing interest in the potential of shared care records to go beyond the better coordination of an individual's care (i.e., by allowing primary and secondary care clinicians to have access to the same information) by embedding linked information (e.g., on the wider determinants of health) into population health management (PHM) schemes. This should support the shift in the balance of care by providing intelligence on where to target more preventive and proactive care.

Again, however, this is huge variation in the maturity of PHM across the country which reflects previous rounds of investment into digital integration. Restricted access to integrated data also limits its potential for shaping strategic intelligence. While this reflects legitimate concerns about privacy and security, it is important that current approaches to information governance are aware of the increasingly sophisticated methods that are available for data anonymisation and protection, lest such systems fall behind the technologies they oversee.

### The regulatory landscape

By finding undiagnosed disease or indicators of clinical deterioration and thereby identifying people who could benefit from early intervention or who require urgent care, an effective “command and control” system that is using AI technology to process millions of data items clearly offers potential to promote population health, improve quality and outcomes, reduce demands on staff time and reduce overall costs (i.e., address the quadruple aim). Given the significant financial pressures facing the NHS, particularly coastal systems serving older demographical areas, slow progress in data integration and orchestration is a lost opportunity cost.

Yet, local health and care systems must negotiate wider institutional challenges to unlocking the potential of DHTs. For example, since 2021, AI that influences clinical decision-making has been classified as “AI as a medical device” (AIaMD), which thus requires regulation by the Medicines and Healthcare products Regulatory Agency (MHRA). While the intention is to create a regulatory framework that is proportionate, precise guidelines about how to demonstrate that AlaMD meets regulatory approval have yet to be published. This creates a somewhat fuzzy area for procurement. Some, usually US-based clinical data platforms such as Orion and CioX are already being procured by some Integrated Care Boards (ICBs, the commissioners of health care services in England) and individual trust providers. These will comply with US federal and state regulations with respect to the development of their policies, procedures, and technology but not necessarily UK regulations. Slow adopter ICBs and providers may be more cautious due to other institutional drivers, such as requirements concerning cyber security, information governance and legal responsibilities. A tendency to prioritise compliance over ambition may be associated with the digital maturity of a health and care ecosystem as well as operational and resource pressures, which give rise to more siloed thinking (55).

NHS procurement is also expected to be informed by robust evidence of e.g., quality, safety, effectiveness and cost-effectiveness. Despite a proliferation of DHTs, few meet these evidential requirements. This lack of evidence probably says more about methodological expectations than of the effectiveness of DHTs themselves. The evidence standards framework (ESF) for digital health technologies published by the National Institute for Health and Care Excellence (NICE) provides a useful guide to standards relating to design, value, performance and deployment and the kind of information that companies developing DHTs needs to collect to meet each standard (56). However, the standards set out for even the lowest tier of technologies, such as smartphone apps and standalone software are quite exacting, particularly for the small and medium sized enterprises (SMEs) that dominate this sector, and which are unlikely to have the capacity to engage in the comprehensive evidence gathering set out in the guidance. This has given rise to an evidence gap, NHS procurers expecting information that many developers are unable to provide. It is common to hear complaints about cold calling by “digital snake oil salesmen” operating in the digital equivalent of an unregulated “Wild West”.

### Cultural resistance

Cultural resistance to digitalisation may stem from a lack of confidence—the digital health competencies expected of health and care professionals are now very wide ranging (57, 58). Staff need to have confidence in the role of technology in delivering high quality, financially viable, resource efficient and sustainable services for their patients. There are also some concerns that, far from reducing demand, digitalisation can cause extra workload. There is not robust evidence for this, in part because of the methodological weakness of studies (59) and it is generally assumed that health and care staff who feel burdened by their digital systems are working with outdated systems that have been poorly designed and implemented. Against this background, an eloquent account by Atal Gawande on how using the relatively new EPIC EPR is potentially contributing to physician burnout is a sobering read (60).

To make the most of home-based DHTs designed to help people stay healthy and independent, citizens require reassurance too. Unfortunately, the very groups (those who are older and/or experiencing deprivation) who are at increased risk of NCDs are also more likely to be digitally excluded. Using data from the 2020 Use of Communication Services survey, an estimated 10% of adults in the UK did not use the internet or have access to it at home. Old age, living alone, having a limiting or impacting condition and being financially vulnerable all significantly contributed to the likelihood of being digitally excluded in terms of use of or access to the internet (61). Broadband coverage levels are also lower in rural areas (no information is available for coastal areas). For example, around one in six rural premises cannot access superfast broadband and over half cannot get an indoor 4G mobile connection on the four main networks. The reliability of digital networks in rural areas is also an issue (62).

### Concerns about environmental impacts

Insofar as digital technologies are expected to reduce travel (e.g., to seek care) and smooth flows of activity within health care facilities, they are generally associated with a reduction in carbon emissions. In fact, the production and disposal of DHTs are not carbon neutral, the raw materials required for wearable technologies, robotics and devices (e.g., iron, aluminium, gold, mercury, cyanide) requiring large mining operations, while a very small proportion of electronic technologies are recycled, raising concerns about e-waste (63). The energy required to store and process very large health datasets is also a concern though again there are many technological solutions to this, from green cloud computing to the use of intelligent building design to e.g., improve cooling and air flow management in large data centres.

## Enablers of digital transformation

### Central guidance and support

In recognition of variations in digital maturity, NHS England (NHSE) committed over £12 m to its Digital Productivity Programme. However, the flagship target to have EPRs in 90 per cent of trusts by the end of 2023 is unlikely to be met. There are concerns that organisational turbulence at the centre, together with cost cutting and the departure of key digital champions have reduced the NHSE's commitment to driving through digital transformation, particularly in areas that need to level up. At the same time, the strong central mandate on productivity savings in the acute sector to balance the books is drawing attention from transformational efforts to shift the balance of care by strengthening prevention (current guidance on which barely mentions the role of digital innovation). The mandate from the centre needs to be more ambitious, retain its focus on digital levelling up and be properly resourced.

### Transparency and shared learning

By providing transparency of what technologies are currently procured by services and what is needed to support local communities, ecosystems can better support digital transformation strategies that address nuanced needs. Challengingly, health services are opaque and heterogeneous with regards to their strategic priority areas for health service delivery and in particular what technologies are in use or might be needed. For example, only sixty percent of ICSs have a digital strategy (64) and most individual service providers fail to openly publish their priority areas and digital transformation agendas. To accelerate digital transformation in coastal communities, a collective effort across the digital health ecosystem is required driven by local priority areas. Common goals are essential to support open innovation ecosystems that leverage the skills and resources of various stakeholders including academia, industry, and government. Standardisation in how this information is conveyed would further support strategic investments in research and technology development activities.

Many learnings relating to the development and deployment of technologies are not shared in traditional academic journals with knowledge held by industry or service providers who embark on internally reported quality improvement programs. If we are to reduce duplication of effort and accelerate digital transformation far greater transparency in digital innovation methodologies and the implementation science driving change are needed, both successes and failures.

### Enhancing digital readiness and architecting a learning health system

To support improved healthcare outcomes and ensure the sustainability of public health systems, data-driven decision-making and continuous learning are essential. The coordination of large scale linked data including patient specific health and wellbeing information, patterns of health service utilisation and the ancillary operational processes and societal costs will accelerate value-based health care. Notwithstanding the fact that many systems in digitally immature coastal and rural areas are at the start of their transformation journey, there may be investments that could yield benefits in terms of both patient outcomes and cost savings. Focusing on prevention (and avoidance of hospital admission) as opposed to the more common approach of supporting hospital discharge through “virtual wards” may be more cost effective in the long run. Given some variation between Integrated Care Systems (ICSs) in the deployment of digital innovation to support discharge vs. avoidable admission, it would be helpful to accrue some evidence of the relative cost-effectiveness of both approaches, a task for NHSE, but not one we suspect would be amenable to available data.

The digital immaturity of coastal communities may prove opportunistic by allowing ICSs to architect the core infrastructure and data needed to effectively support value-based health care e.g., the routine deployment of preference-based patient reported outcome measures to support cost-effectiveness analysis and help to better demonstrate return on investment over a longer time horizon. Additionally, non-direct data is needed to contribute to evidence-based innovation and decision-making such as carbon emission. As part of the NHS net zero carbon agenda, digital transformation with the UK is inextricably linked with all construction and supply chain partners who will need to demonstrate their carbon impact and strategies to proactively reduce emissions by 2030, reaching net zero by 2045 (65). Greater transparency in the environmental impact of the health services ecosystem through disaggregated indices is therefore essential to facilitate accuracy in accurate carbon accounting and adequately demonstrate emissions reductions as a result of digital transformation.

If ICSs are to invest in the use of DHTs for prevention, early diagnosis and care within the home, they need to set up orchestration layers that draw data from various platforms into a single “command and control” centre (CCC) in which clinical teams identify triggers for intervention, an approach that should be increasingly supported through AI. Some coastal ICSs are doing this through pilots. However, integrating data, negotiating information governance barriers etc., is proving to be highly labour intensive. Perhaps more attention needs to be paid to understanding why such an obviously useful approach is so difficult to enact in practice and which institutional barriers need to be overcome to make better use of shared data and e.g., AI (an unexplored territory with variation between different health care systems).

Finally, active steps need to be taken to ensure that socio-economic variations in digital inclusion do not exacerbate socio-economic inequalities in health. This is a particular issue in coastal areas due to older demographic populations (age being a key determinant of digital exclusion), variable broadband on the periphery and fears that, in responding to the current cost of living crisis, financially challenged groups are more likely to prioritise basic needs than internet subscriptions. In many such areas, the Voluntary, Community and Social Enterprise Sector (VCSE) is playing a critical role in addressing disparities in access to technology, skills, confidence and systems, in some cases such as Devon, through financial support from ICBs. Imaginative solutions are also being developed with respect to promoting access through e.g., community buildings.

In short, the technological solutions are there, the vision and infrastructure are not. ICSs/ICBs are increasingly looking to US-based companies to provide such technological solutions. Whether such solutions fit into existing NHS systems or support the UK Department for Science, Innovation and Technology's remit to promote digital innovation as an economic driver is debatable.

### Creating digital living labs

It is generally agreed that, to address the gap between the development of DHTs and their implementation, it is helpful to support partnerships between industry, academia and end users (whether health and care staff or citizens). There is international interest in potential of “living labs” to this end. Defined as open innovation systems where end users and other stakeholders are involved in the exploration, co-creation and evaluation of solutions in realistic circumstances (66), living labs offer test-beds for co-creation (co-design by users and producers), exploration (discovering emerging usages, behaviours and market opportunities), experimentation (implementing live scenarios within communities of users) and evaluation (67). Living labs could address several barriers identified above, opportunities for co-design ensuring better organisational fit and potentially reducing cultural resistance, while embedding evaluation supports improvements in evidence-based digital health. Indeed, as there are international networks of living labs, such as the Australian Living Lab Innovation Network and the European Network of Living Labs, there is the potential to carry out international evaluations of DHTs, opening up the market for innovators as well as reducing replication of effort. To date, however, cross-cultural differences in research design remain a challenge (68), raising questions about methodological consistency.

There are some digital health living labs in the UK, some are *in situ* facilities within homes and health services. More commonly however, they are off-site and established by academic institutions or community innovation hubs. These are randomly distributed and their creation has tended to be dependent on the efforts of local champions. Mobile digital health living-labs are a nascent concept. Much like mobile medical vans, these facilities are intended to overcome barriers to digital innovation and adoption by meeting people where they are—a particular model that would benefit coastal communities for all the geographical and sociocultural factors previously discussed.

Living labs are a key lever for innovation and adoption in a digital health ecosystem, and there is a strong case for UK Research and Innovation (UKRI) bodies, NHS England and individual ICBs to explore opportunities to rolling out labs more systematically. To this end, it would be helpful to think outside the box with respect to key system partners. For example, social housing organisations provide a unique opportunity to develop, test and evaluate DHTs within the home and village hubs (e.g., community halls) to provide dispersed coastal and rural communities with an opportunity to explore technology.

### Understanding evidence

The limitations of the current evidence base in digital health are one factor behind their relatively slow adoption in the UK. Small and Medium sized Enterprises (SMEs) are particularly challenged with respect to meeting the evidential requirements of commissioners (e.g., evaluation of usability, effectiveness and cost-effectiveness of DHTs). Commissioners themselves may be overly reliant on randomised control trial (RCT) evidence, although RCTs are not often appropriate for rapidly developing DHTs. Thus, there is a need to provide generic training for SMEs on how to develop their value proposition and produce evidence of how their product fits into the system and whether it delivers an effective and cost-effective solution as well as key business and accreditation skills (e.g., how to do light-touch market research analysis; product-market fit, accreditation requirements etc.). At the same time, clinicians require reassurance about clinical safety and accountability, while managers could benefit from training in the more agile assessment of DHTs. There are existing providers of such training in the UK. However, there are some concerns that their costs are too high for smaller SMEs. Again, local health and care systems could benefit from tapping into international networks such as the European Connected Health Alliance Group (ECHAlliance Group), many members of which are training providers. Importantly, part of the NHS net zero carbon agenda, digital transformation with the UK is inextricably linked with all construction and supply chain partners who will need to demonstrate their carbon impact and strategies to proactively reduce emissions by 2030, reaching net zero by 2045 (65). Greater transparency in the environmental impact of the health services ecosystem through disaggregated indices is therefore essential to facilitate accuracy in accurate carbon accounting and adequately demonstrate emissions reductions and return on investment.

### Digital skills training

If health and care practice is to be increasingly supported by new technologies, much more needs to be done to upskill the current workforce and prepare future professionals. Health Education England provides several NHS Digital Academy learning programmes, including a Chief Nursing Information Officer (CNIO) masterclass series and there has been a drive to appoint a chief nursing information officer (CNIO) in every organisation to help support the digital agenda. With respect to health informatics, nursing appears to be further ahead than other professions (69), perhaps because nurses appear to be taking on the lion's share of responsibility for entering and monitoring digital data (70). However, digital innovation influences all areas of medicine, nursing and allied health professions and the strong focus to date on health informatics (58) is not preparing professionals for working in a digitised health and care system (71). Again, as much training is experiential, staff working in digitally immature ecosystems are less likely to become confident and skilled than those located in digital exemplars.

Within undergraduate courses, there is significant variation between UK universities in the extent to which digital training is embedded into medical, nursing and allied health profession education. There is currently no central guidance (e.g., from the Academy of Medical Royal Colleges and the General Medical Council) on how to embed digital competence into the medical curriculum, let alone how to train doctors to manage the rapid pace of technological innovation. Published in 2019, the Topol Review (72) presented a compelling vision for preparing the healthcare workforce to deliver the digital future. Going beyond health informatics to consider the role of genomics, artificial intelligence, digital medicine and robotics, the report's recommendations are currently being explored in curriculum development plans and are likely to provide a much-needed driver for the improvement of both undergraduate and continuing professional education.

Importantly, given the sustained proliferation of health technology, multiple methods must be offered to professionals to adequately support skills development. Embedding opportunities for digital awareness and skills development within traditional training programmes as a cross cutting theme as opposed to a bolt on module is needed to adequately support the digital readiness of professionals whereby technologies are seen as an enabler of routine care. Real-world case studies and opportunities for practical exploration of technology such as through virtual and physical technology libraries will enable continuous exploration and learning while more bespoke postgraduate programmes are needed to support specialisation within the digital roles such as digital hospital in the home programs.

Given the rapid evolution of technology and the considerable variation in personal skills, competencies, career trajectories and variation in what technology is available in any given setting training is more likely to occur outside of traditional programmes such as through short courses. The development of micro competences and the scaffolding of learning must ideally be CPD accredited wherever relevant to demonstrate professional growth and pave the way for new career opportunities. Irrespective of delivery type, it is widely acknowledged that digital technologies must foremost be designed to be intuitive in their use thus eliminating skills-related barriers to their adoption and optimisation.

## Conclusion

In this paper, we have noted the challenges facing coastal health and care systems in England, which are characterised by a “coastal excess” in the prevalence of disease and risk factors and evidence of a service deficit. The latter may stem from a system of resource allocation which may disadvantage areas serving older demographic populations and that does not adjust for the difficulties systems serving peripheral and dispersed populations face in achieving economies of scale. We have made the case that digital transformation offers the potential to shift the balance of care from reactive, expensive hospital management to prevention, early diagnosis and management within the home. However, coastal areas are also more likely to be digitally immature, raising concerns that variations in digital maturity may be exacerbating coastal health inequalities.

Some of the barriers to digital transformation require a response from central bodies to e.g., level up digital maturity, a role for NHS England; streamline routes to standards compliance, regulatory approval (NICE, MHRA and NHSE); and provide clear guidance as to how undergraduate and continuing education curricula can better prepare the workforce for maximising the potential of DHTs, a role that is being assumed by Health Education England but that also requires involvement from the professional bodies. In other cases, health service managers within ICBs, ICSs and local trusts can play an important role in accelerating digital transformation. We have proposed that priority areas should include investment into orchestration layers in particular to allow remotely monitored data to be integrated into single platforms and analysed so as to allow early diagnosis and intervention to take place at a large scale. Rolling out a more ambitious programme of digital living labs is also within the gift of local organisations. Focusing test-bed opportunities around people's homes and local communities would again facilitate a shift in emphasis from the hospital to the role that should be played by wider partners in an *integrated care system*.

In the scope of this paper, we have only been able to touch upon the enormous potential of DHTs to promote better population health, reduce health inequalities, improve the experience and outcomes of care, reduce demands on the health and care workforce, and ultimately reduce costs. Yet, the NHS, particularly in coastal and rural areas, remains years behind countries such as the USA in its deployment of DHTs, particularly those that allow the remote monitoring of patients. We have outlined some of the complex reasons why the NHS has struggled to embed digital health innovations and offered some solutions. What is essentially required, however, is the vision and the willingness to embrace a digital future. We hope that this paper can make a contribution towards winning the hearts and minds of the policy leaders and health service managers who can effect such change.
